# Supplementary figures and images for: Diversity analysis of gut microbiota in osteoporosis and osteopenia patients
Source: PeerJ. 2017 Jun 15;5:e3450. doi: 10.7717/peerj.3450 (PMC5474093; doi:10.7717/peerj.3450)

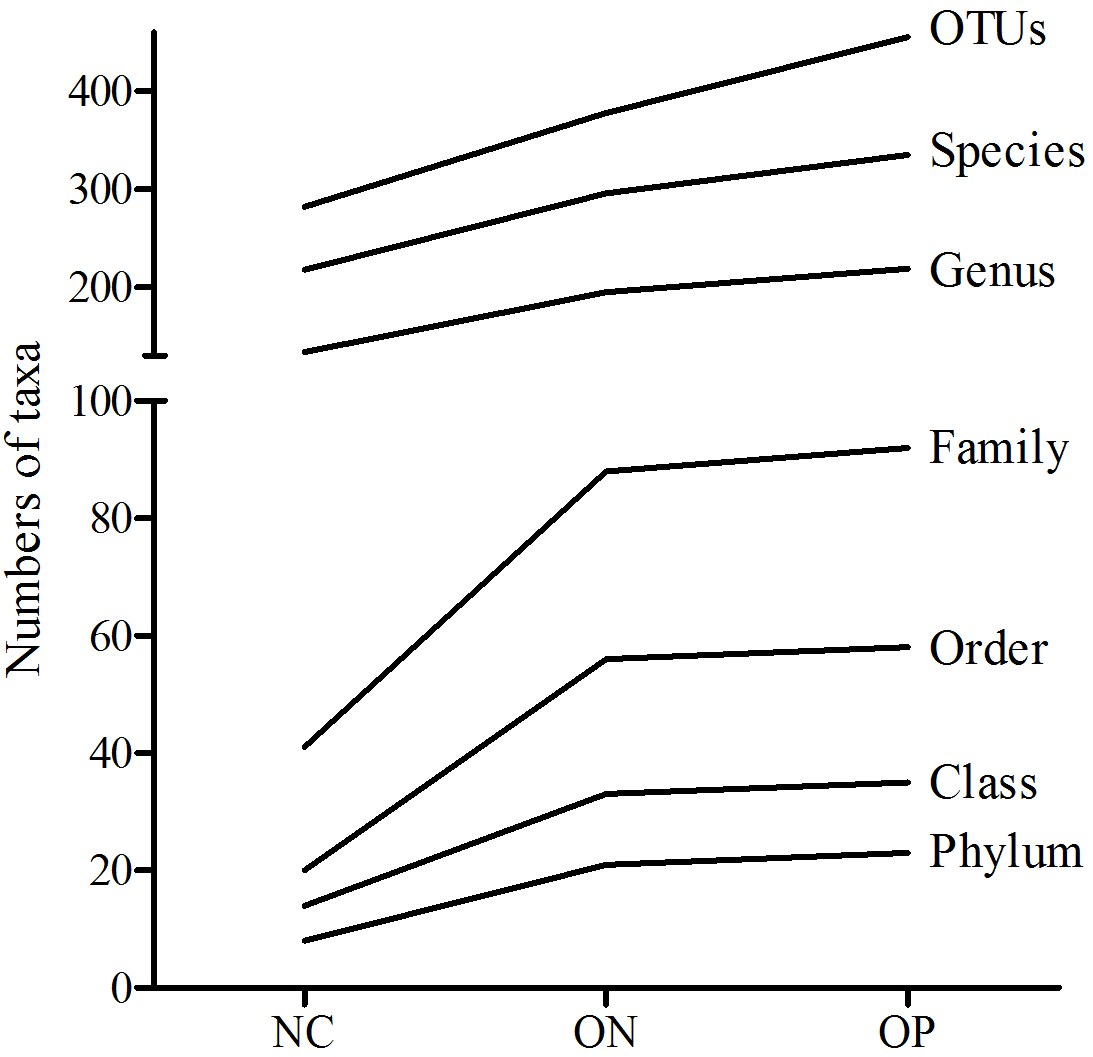

Supplement: Figure S1 [file peerj-05-3450-s001.png]

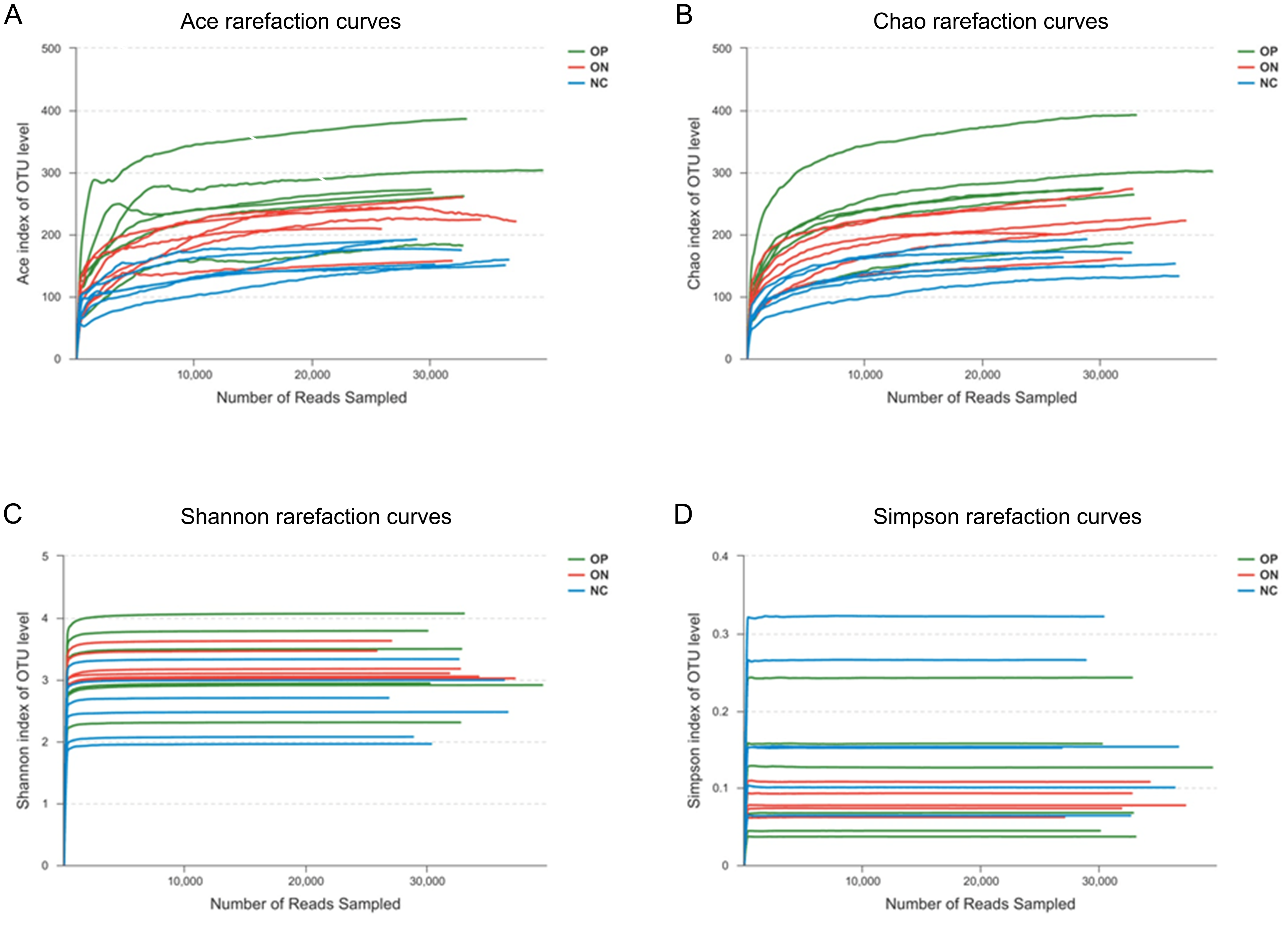

Supplement: Figure S2 [file peerj-05-3450-s002.png]

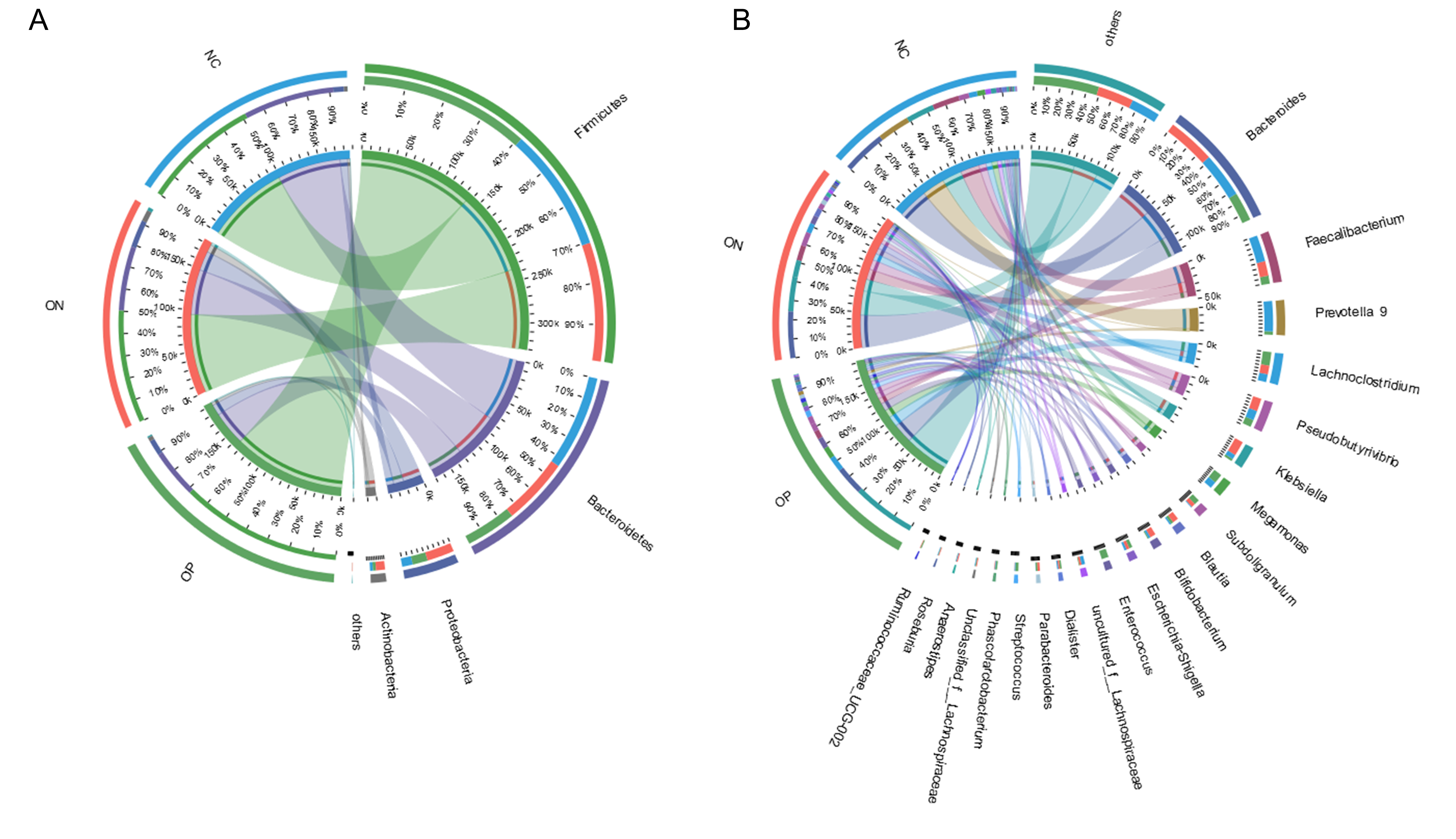

Supplement: Figure S3 — (A) Collinearity diagram at phylum level. (B) Collinearity diagram at genus level. [file peerj-05-3450-s003.png]
