# Supplementary material for: Diversity analysis of gut microbiota in osteoporosis and osteopenia patients
Source: PeerJ. 2017 Jun 15;5:e3450. doi: 10.7717/peerj.3450 (PMC5474093; doi:10.7717/peerj.3450)
Supplement: Table S1 [file peerj-05-3450-s004.doc]

**Table S1 Illumina sequence information of each sample.**

| Sample\Info | Seq_num | Base_num | Mean_length | Min_length | Max_length |
| --- | --- | --- | --- | --- | --- |
| NC1 | 40711 | 17890180 | 439 | 270 | 452 |
| NC2 | 31129 | 13501857 | 434 | 400 | 451 |
| NC3 | 43906 | 19087002 | 435 | 338 | 452 |
| NC4 | 34696 | 15257974 | 440 | 420 | 452 |
| NC5 | 31910 | 14136797 | 443 | 411 | 453 |
| NC6 | 42075 | 18179118 | 432 | 338 | 464 |
| ON1 | 30238 | 12996413 | 430 | 381 | 465 |
| ON2 | 44314 | 19508304 | 440 | 358 | 452 |
| ON3 | 42600 | 18385245 | 432 | 346 | 455 |
| ON4 | 37892 | 16720433 | 441 | 368 | 473 |
| ON5 | 36209 | 15707157 | 434 | 403 | 452 |
| ON6 | 34623 | 15092595 | 436 | 203 | 453 |
| OP1 | 43489 | 18762550 | 431 | 358 | 486 |
| OP2 | 40925 | 17746681 | 434 | 360 | 462 |
| OP3 | 37909 | 16673545 | 440 | 384 | 492 |
| OP4 | 38323 | 16979717 | 443 | 382 | 453 |
| OP5 | 38564 | 16574675 | 430 | 366 | 452 |
| OP6 | 44719 | 19722882 | 441 | 327 | 454 |
