# Supplementary material for: Diversity analysis of gut microbiota in osteoporosis and osteopenia patients
Source: PeerJ. 2017 Jun 15;5:e3450. doi: 10.7717/peerj.3450 (PMC5474093; doi:10.7717/peerj.3450)
Supplement: Table S2 [file peerj-05-3450-s005.doc]

**Table S2 Estimators of alpha diversity of each sample.**

| Sample\  Estimators | ace | chao | shannon | 1/simpson |
| --- | --- | --- | --- | --- |
| NC1 | 177.536 | 173 | 3.334414 | 15.57438 |
| NC2 | 187.7208 | 162.0769 | 2.702705 | 6.57527 |
| NC3 | 152.2616 | 158 | 2.994291 | 9.938678 |
| NC4 | 151.9588 | 147.3529 | 1.961493 | 3.106246 |
| NC5 | 191.4899 | 191.3333 | 2.078802 | 3.767656 |
| NC6 | 160.0238 | 135.3333 | 2.478391 | 6.523072 |
| ON1 | 205.3185 | 196.6071 | 3.458275 | 15.48419 |
| ON2 | 227.361 | 228.4737 | 3.021578 | 12.8974 |
| ON3 | 218.5306 | 220.0588 | 3.03805 | 9.204628 |
| ON4 | 258.2515 | 268.05 | 3.177343 | 10.74483 |
| ON5 | 157.6121 | 161.0769 | 3.105577 | 13.60082 |
| ON6 | 243.1752 | 247.625 | 3.623966 | 16.05652 |
| OP1 | 260.3745 | 265 | 3.492852 | 14.71432 |
| OP2 | 390.3002 | 397.1818 | 4.074678 | 27.08046 |
| OP3 | 204.614 | 192.4615 | 2.311026 | 4.118803 |
| OP4 | 266.0439 | 271.2917 | 2.937315 | 6.344775 |
| OP5 | 271.3796 | 274.4545 | 3.785153 | 22.34237 |
| OP6 | 304.2477 | 303.0278 | 2.914457 | 7.90164 |
